# Supplementary material for: Regulation of Microglial Signaling by Lyn and SHIP-1 in the Steady-State Adult Mouse Brain
Source: Cells. 2023 Sep 28;12(19):2378. doi: 10.3390/cells12192378 (PMC10571795; doi:10.3390/cells12192378)
Supplement: Supplementary file 1 [file cells-12-02378-s001.zip › cells-2623351-supplementary.pdf]

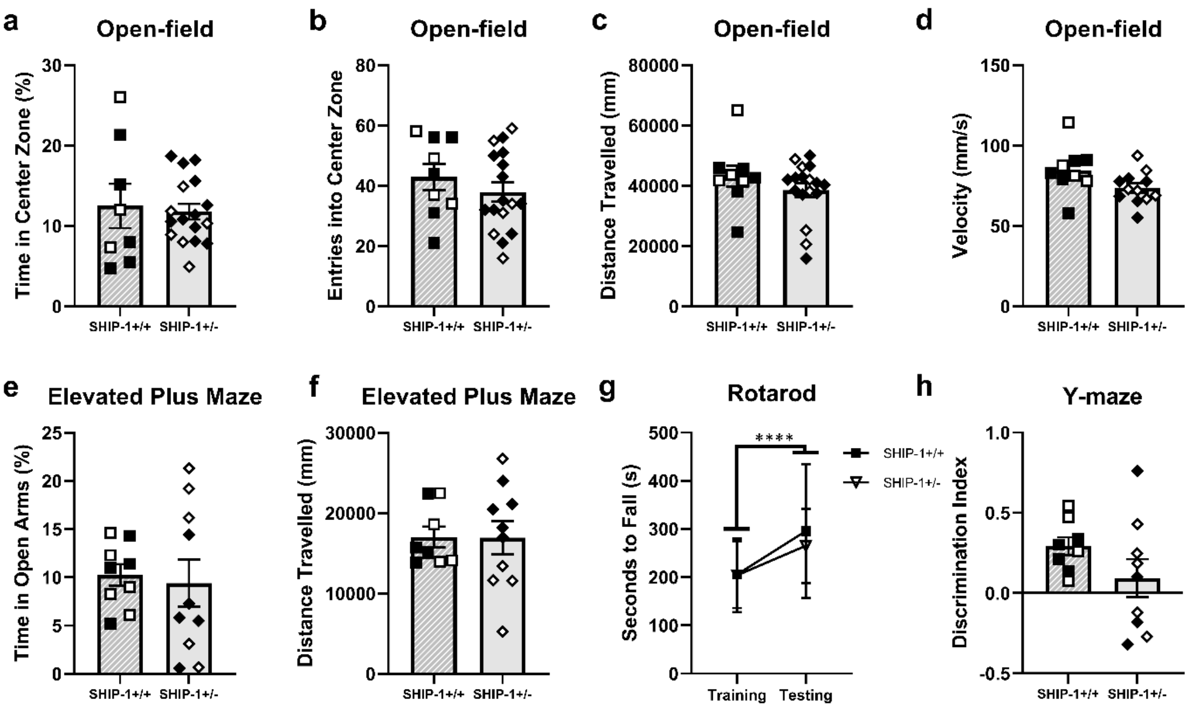


**Supplementary Figure S1.** Heterozygous SHIP-1+/− mice exhibit no differences in behavior compared to SHIP-1+/+ mice. (a) Time spent in center zone; (b) entries into center zone; (c) distance traveled; and, (d) speed in open-field tests (n= 9 SHIP-1+/+ and 17 SHIP-1+/− mice). (e) Time spent in open arms; and, (f) total distance traveled during Elevated Plus Maze test (n= 9 SHIP-1+/+ and 10 SHIP-1+/− mice). (g) Time (sec) spent on rotarod before falling during consecutive training and testing days (n= 9 SHIP-1+/+ and 10 SHIP-1+/− mice). (h) Discrimination index calculated from time spent in novel arm against time spent in familiar arm during Y-maze test (n = 8 SHIP-1+/+ and 9 SHIP-1+/− mice). Females = solid, males = open. Unpaired t-test and Two-way ANOVA ****p<0.0001.

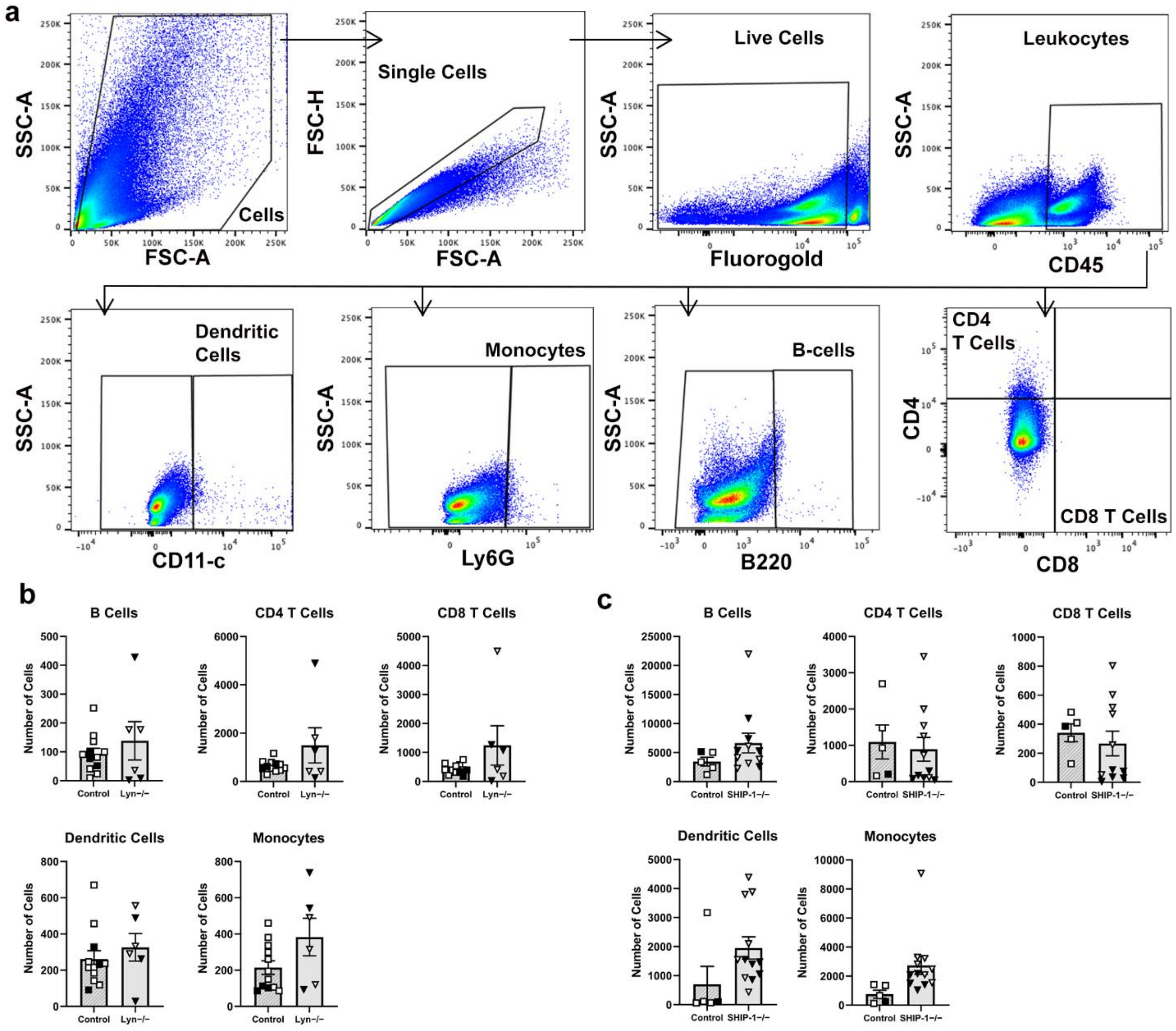


**Supplementary Figure S2.** The immune cell composition in the steady-state brains of Lyn−/− and SHIP-1−/− mice is unchanged. (a) Gating strategy to identify cell populations in digested whole brain tissue from Lyn−/− and SHIP-1−/− mice, and their respective controls. Flow cytometric quantitation of the indicated immune cell subsets in digested whole brain tissue from (b) Lyn−/− mice (n = 6 Lyn−/− and 12 control mice); and, (c) SHIP-1−/− mice (n = 12 SHIP-1−/− and 5 control mice). Females = solid, males = open.

**Supplementary Table S1. Distribution of animals used in each set of experiments.**

| **Distribution of Lyn−/− mice used in study** | | |
|---|---|---|
| Procedures | Genotype | |
| | Control (C57BL/6) | Knockout (Lyn-/-) |
| Behavior | n=18 (m=12, f=6) | n=19 (m=10, f=9) |
| Histology | n=7 (m=4, f=3) | n=6 (m=3, f=3) |
| Gene Expression | n=6 (m=3, f=3) | n=6 (m=6, f=0) |
| Flow Cytometry | n=11(m=8, f=3) | n=11 (m=6, f=5) |
| Cytokine Flow Cytometry | n=10 (m=5, f=5) | n=10 (m=3, f=7) |
| **Distribution of SHIP-1−/− mice used in study** | | |
| Procedures | Genotype | |
| | Control (SHIP-1+/+ or SHIP-1+/−*) | Knockout (SHIP-1−/−) |
| Behavior | n=8 (m=4, f=4) | n=23 (m=9, f=14) |
| Histology | n=8 (m=5, f=3) | n=8 (m=4, f=4) |
| Gene Expression | n=5 (m=5, f=0) | n=6 (m=4, f=2) |
| Flow Cytometry | n=10 (m=7, f=3) | n=16 (m=9, f=7) |
| Cytokine Flow Cytometry | n=9 (m=5, f=4) | n=8 (m=1, f=6) |

* Littermate SHIP-1+/+ mice were used as controls for all experiments except for flow cytometry analyses, where littermate SHIP-1+/− mice were used as controls.

**Supplementary Table S2. Antibodies used for flow cytometry.**

| Marker | Clone | Channel | Conc. | Catalogue | Company |
|---|---|---|---|---|---|
| CD16/CD32 Fc block | - | - | 1:640 | 553141 | Produced in-house |
| Fluorogold (live-dead stain) | - | BUV525 | 1:1000 | Sc-358883A | Santa Cruz Biotechnology |
| **Brain Analyses** | | | | | |
| IBA-1 | 1022-5 | FITC | 1:500 | Ab15691 | Abcam |
| CX3CR1 | SA011F11 | PerCP Cy5.5 | 1:500 | 149010 | BioLegend |
| CD45 | 30-F11 | BUV395 | 1:1000 | 564279 | BD Bioscience |
| TREM2 | Polyclonal | Biotinylated | 1:300 | RDSBAF1729 | Labome (R&D Systems) |
| CD86 | GL1 | BV421 | 1:500 | 564198 | BD Bioscience |
| CD206 | IMR5D3 | Biotinylated | 1:400 | MA5016869 | Invitrogen |
| TMEM119 | V3RTGOsz | PE-Cy7 | 1:500 | 25-6119-82 | Invitrogen |
| CD40 | 3/23 | APC | 1:500 | 558695 | BD Bioscience |
| CD4 | GK1.5 | FITC | 1:600 | 553729 | BD Bioscience |
| CD14 | 5A2-8 | PerCP Cy5.5 | 1:500 | 45-0141-82 | |
| CD8a | 53-6.7 | APCe780 | 1:500 | 47-0081-82 | eBioscience |
| CD11c | HL3 | PE | 1:500 | 553802 | BD Bioscience |
| B220 | RA3-6B2 | PE-Cy7 | 1:500 | 25-0452-82 | eBioscience |
| F4/80 | T45-2342 | BUV737 | 1:800 | 748283 | BD Bioscience |
| CCR2 | SA203G11 | BV421 | 1:500 | 150605 | Biolegend |
| Ly6G | 1A8 | BV510 | 1:500 | 127633 | Biolegend |
| CD11b | M1/70 | PE-Cy7 | 1:500 | 25-0112-82 | Invitrogen |
| CD22.2 | G155-178 | Biotinylated | 1:500 | 553382 | eBioscience |
| **Spleen (Single Stain Set-up Controls)** | | | | | |
| B220 | RA3-6B2 | FITC | 1:1000 | 553088 | BD Bioscience |
| B220 | RA3-6B2 | PerCP Cy5.5 | 1:1000 | 552771 | BD Bioscience |
| B220 | RA3-6B2 | APC | 1:1000 | 553092 | BD Bioscience |
| B220 | RA3-6B2 | APCe780 | 1:1000 | 47-0452-82 | eBioscience |

| | | | | | |
|---|---|---|---|---|---|
| B220 | RA3-6B2 | PE | 1:1000 | 553090 | BD Bioscience |
| B220 | RA3-6B2 | PE-Cy7 | 1:1000 | 25-0452-82 | eBioscience |
| CD19 | 1D3 | BV450 | 1:400 | 48-0193-82 | eBioscience |
| B220 | RA3-6B2 | Biotinylated | 1:1000 | 553086 | BD Bioscience |
| **Secondary** | | | | | |
| - | Streptavidin | BV711 | 1:1000 | 563262 | BD Bioscience |
| **Intracellular Cytokine** | | | | | |
| TNF-α | Monoclonal | BV450 | 1:100 | 48-7321-82 | Thermo Fisher Scientific |
| IgG Control | - | PE | 1:100 | 554680 | BD Bioscience |

**Supplementary Table S3. Gene assays used for gene expression analyses.**

| Marker | Gene Name | Gene Assay ID (Thermo Fisher) |
|---|---|---|
| Chemokine (C-C Motif) Ligand 2 (CCL2) | *Ccl2* | Mm00441242_m1 |
| CD16 | *Fcgr3* | Mm00438882_m1 |
| CD68 | *Cd68* | Mm03047343_m1 |
| CD86 | *Cd86* | Mm00444540_m1 |
| CD206 | *Mrc1* | Mm01329362_m1 |
| Interleukin 1 beta (IL-1β) | *Il1b* | Mm00434228_m1 |
| Ionized Calcium-binding Adapter Molecule 1 (IBA-1) | *Aif1* | Mm00479862_g1 |
| Glial Fibrillary Acidic Protein (GFAP) | *Gfap* | Mm01253033_m1 |
| Lyn | *Lyn* | Mm01217481_m1 |
| Src Homology 2 Domain Containing Inositol Polyphosphate 5-phosphate 1 (SHIP-1) | *Inpp5d* | Mm00494963_m1 |
| TMEM119 | *Tmem119* | Mm00525305_m1 |
| TREM2 | *Trem2* | Mm04209424_g1 |
